# Unveiling the Significance of Graphene Nanoplatelet (GNP) Localization in Tuning the Performance of PP/HDPE Blends

**DOI:** 10.3390/ma17225673

**Published:** 2024-11-20

**Authors:** Reza Salehiyan, Ali A. El-Samak, Milad Kamkar, Elnaz Erfanian, Stephen A. Hodge, Uttandaraman Sundararaj, Tony McNally

**Affiliations:** 1 School of Computing, Engineering and the Built Environment, Edinburgh Napier University, Edinburgh EH10 5DT, UK; 2 International Institute for Nanocomposites Manufacturing (IINM), WMG, University of Warwick, Coventry CV4 7AL, West Midlands, UK; ali.el-samak@warwick.ac.uk (A.A.E.-S.); t.mcnally@warwick.ac.uk (T.M.); 3 Department of Chemical Engineering, Waterloo Institute for Nanotechnology, University of Waterloo, 200 University Avenue West Waterloo, Waterloo, ON N2L 3G1, Canada; milad.kamkar@uwaterloo.ca; 4 Department of Chemical and Petroleum Engineering, University of Calgary, Calgary, AB T2N 1N4, Canada; elnaz.erfanian@ucalgary.ca (E.E.); ut@ucalgary.ca (U.S.); 5 Versarien Plc, Longhope GL17 0QZ, Gloucestershire, UK; stephen.hodge@versarien.co.uk

**Keywords:** polymer blends, graphene nanoplatelets, blending sequence, crystallization, high density polyethylene, polypropylene

## Abstract

High-density polyethylene (HDPE) and polypropylene (PP) blends are widely used in industries requiring mechanically durable materials, yet the impact of processing parameters on blend performance remains underexplored. This study investigates the influence of blending sequence and screw speed on the properties of blends of HDPE and PP filled with 1.25 wt.% graphene nanoplatelets (GNPs). Changes in crystallization behaviour, tensile strength, and viscoelastic responses with blending sequence are studied. The addition of GNP increases the crystallization temperature (T_c_) of PP in the PE/PP blend by 4 °C when GNP is pre-mixed with PE to form (PE+GNP)/PP blends. In contrast, when GNP is pre-mixed with PP to create (PP+GNP)/PE blends, the T_c_ of PP rises by approximately 11 °C, from 124 °C for the neat PE/PP blend to 135 °C. On the other hand, the T_c_ of PE remains unchanged regardless of the blending sequence. XRD patterns reveal the impact of blending regime on crystallinity, with GNP alignment affecting peak intensities confirming the more efficient interaction of GNPs with PP when premixed before blending with PE, (PP+GNP)/PE. Tensile moduli are less sensitive to the changes in processing, e.g., screw speed and blending sequence. In contrast, elongation at break and tensile toughness show distinct variations. The elongation at the break of the (PP+GNP)/PE blend decreases by 30% on increasing screw speed from 50 to 200 rpm. Moreover, the elongation at the break of (PE+GNP)/PP prepared at 100 rpm is ~40% higher than that of the (PP+GNP)/PE. (PE+GNP)/PP displays a ‘quasi-co-continuous’ morphology linked to its higher elastic modulus G′ compared to that of the (PP+GNP)/PE blend. This study highlights the importance and correlation between processing and blend properties, offering insights into fine-tuning polymer composite formulation for optimal performance.

## 1. Introduction

Polymer blends, the combination of two or more polymers, continue to be a versatile class of material that offers a diverse range of properties by combining the distinct characteristics of the individual components. Polyolefin blends, particularly polyethylene (PE) and polypropylene (PP) blends, have received significant attention due to their wide industrial applications and exceptional mechanical properties [1]. PE is flexible and tough and, when combined with PP, it results in a blend with higher stiffness and heat resistance, providing a balanced set of properties [1,2]. The successful synthesis of polyethylene (PE) and polypropylene (PP) blends has led to a diverse range of applications, from packaging materials to automotive components. Due to their immiscibility resulting from large interfacial tensions, various morphologies can be achieved. Therefore, refining these morphologies into more stable and homogeneous structures is necessary to obtain uniformly enhanced performance. The incorporation of nanoscale additives into polymer matrices has further extended the field, allowing for the fine-tuning of blend properties. Among the various nanoparticles, 2D graphene nanoplatelets (GNPs) have emerged as a promising candidate, due to their exceptional mechanical, electrical, and thermal properties. The unique combination of mechanical reinforcement and multifunctional properties may allow for its use in innovative applications, including lightweight composites, conductive coatings, and advanced sensors. With the rapid growth of the electric vehicle and autonomous automotive sectors, materials that integrate both mechanical resilience and effective electromagnetic interference shielding are becoming increasingly essential. Rigorous safety standards, including ISO 11452 [3] and CISPR 25 [4], emphasize the need for materials that can meet both structural demands and electromagnetic compatibility requirements, making such composites highly suitable for emerging automotive technologies. References [5,6,7,8,9,10,11,12] are referred to as a class of stacked graphene layers with lateral dimensions between 100 nm and 100 µm and thickness ranging from 1 to 3 nm [13]. In the field of polymer composites, the method of incorporating nanoparticles can significantly impact the final material properties [14,15]. The order of nanoparticle addition during blending, as well as the overall processing conditions, plays a pivotal role in governing the extent of nanofiller dispersion, distribution, and interaction with the polymer matrix. A carefully designed sequence ensures optimal interaction between the nanoparticles and polymer, influencing composite morphology and overall performance [15,16]. The manipulation of blend morphologies through the incorporation of nanoparticles offers a powerful strategy to engineer material properties [17]. In addition, nanoparticles act as nucleation sites for crystallization, influencing crystal growth and the orientation of the polymer matrix [10]. This, in turn, impacts mechanical strength, thermal conductivity, and electrical behaviour [2,18]. Crystallization, a key aspect of polymer behaviour, can significantly affect the properties of immiscible polymer blends. The presence of nanoparticles, such as GNPs, can alter the crystallization kinetics and morphology, leading to enhanced crystallization rates [19]. This interplay between crystallization, morphology, and the inclusion of nanoparticles can either enhance or hinder the final material properties, depending on the specific application and desired outcomes. The importance of careful control over processing conditions cannot be overstated. Achieving optimal material properties requires a deep understanding of the relevant processing parameters, such as temperature, applied shear, and extensional forces, mixing sequence and residence time. Neglecting these factors can lead to poor dispersion, inadequate interfacial interactions, and compromised mechanical performance [20]. To harness the full potential of polymer blends and their composites, it is essential to fine-tune processing conditions to ensure consistent and reproducible results. By controlling the dispersion and arrangement of nanoparticles, it becomes possible to tailor the balance between stiffness, toughness, and other properties, opening new avenues for advanced materials. One of the critical processing conditions that significantly influences the properties of polymer composites is the blending sequence [16,21,22]. The blending sequence refers to the order in which components are mixed during the processing stages. This seemingly procedural aspect has profound implications for the final properties of the composite. For instance, the sequence in which carbon nanotubes (CNTs) were introduced to polylactic acid/polyvinylidene fluoride (PLA/PVDF) blends resulted in variations in electrical conductivity, dielectric properties, and electromagnetic interference shielding (EMI) properties [16]. Therefore, the interactions between GNPs and the polymer phases are influenced by this sequence, which, in turn, can dictate composite properties. It should be noted that the sequence could play a more effective role if there is thermodynamic affinity between the GNP with one of the phases [23,24]. This would stimulate migration and the re-distribution of the GNP within the bulk polymer blend.

Another critical processing parameter is the screw speed employed during extrusion-based mixing. Beyond mechanical mixing, the mixing speed affects the kinetics of dispersion, intercalation, and the orientation of nanofillers within polymer matrices [25,26,27]. Ock et al. [27] reported a morphology change in PLA/natural rubber/Cloisite 20A blends when mixing speed was increased from 30 to 50 and eventually 100 rpm. The change in morphology was associated with an increase in elastic G′ and loss moduli Gʺ at low frequencies. Thus, controlling the screw speed can facilitate the efficient intercalation of GNPs into the polymer matrix, influencing composite morphology, crystallization kinetics, and overall mechanical properties. Nevertheless, an excessive screw speed can lead to shear-induced degradation, emphasizing the delicate balance required to optimize dispersion without compromising the properties of the polymers. Despite these advancements, understanding how specific processing conditions, namely, blending sequence and screw speed, affect the dispersion of GNPs within immiscible polymer blends like HDPE/PP, especially at low nanofiller loadings, is critical. While blend composition, including the proportion of polymer phases and nanofillers, significantly influences the properties of the composite, the current research interest focuses on the relationship between processing conditions and the final properties of composites made from polymers and GNPs. Previous studies conducted by Tu et al. [28,29] have indicated that graphene and thermally reduced graphene oxide (TRG) preferentially localize in polyethylene (PE) rather than in polypropylene (PP), with theoretical estimations yielding a wetting coefficient of −2.8 for this system. Hence, these nanoparticles would migrate from PP to PE if sufficient mixing time is provided. They also found that a (50/50) PE/PP blend remains electrically insulating below 2 wt.% of (TRG) due to the undeveloped interfacial localization of TRG when it was pre-mixed with PP for 2 min prior to blending with PE for another 5 min to stimulate migration. This was regardless of the sufficient mixing time employed. This reiterates the importance of mixing sequence, mixing time, and shear intensity in developing morphologies for optimal properties. Therefore, in this study, we aim to adapt these parameters to a more industrially viable route using extrusion and injection moulding processing. We seek to explore how the shear rates employed during processing can affect GNP dispersion by implementing different screw speeds and blending sequences, focusing primarily on how crystallization can highlight changes in dispersion. By systematically investigating the impact of blending sequence and screw speed on the crystallization behaviour, tensile strength, and rheological properties of a (50/50) HDPE/PP blend with a low GNP loading of 1.25 wt.%, we aim to contribute valuable knowledge that can guide the development of advanced materials with tailored properties for specific industrial applications.

## 2. Materials and Methods

The HDPE used in this study was Hostalen ACP 5831 D, provided by LyondellBasell, having a density of 0.953 g/cm^3^ and a melt flow rate (MFR) of 0.25 g/10 min at 190 °C and 2.16 kg. The PP was PP1063L1 from ExxonMobil^TM^, with a density of 0.9 g/cm^3^ and an MFR of 8 g/10 min at 230 °C and 2.16 kg. The GNPs, trade name, Nanene-002, were kindly supplied by Versarien. (Gloucestershire, UK), with approximately 91% of the sample having ≤10 platelets and lateral dimensions of up to 26.5 μm. These GNP powders were utilized as received. Detailed information on the GNPs used in this study is listed in Table 1.

**Scheme 1:**
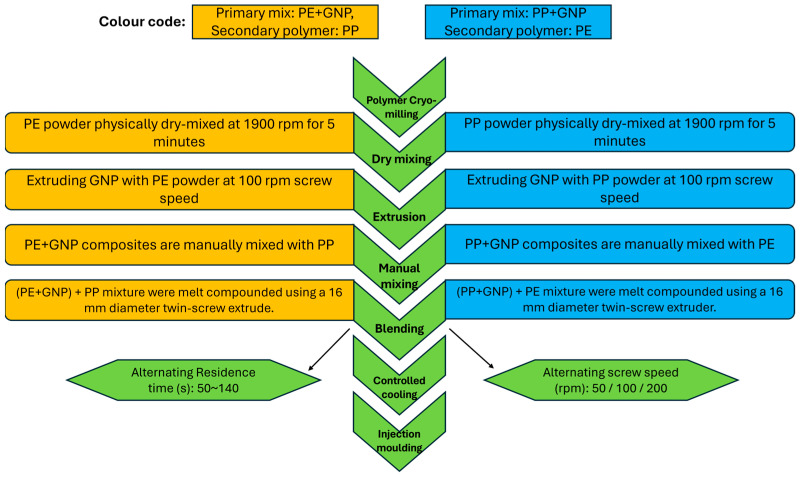
The chart illustrating the entire preparation flow for the composites.

The entire preparation steps are illustrated in Scheme 1. Both PE and PP were firstly cryo-milled to (µm-sized) powders to ensure optimal drying and melt mixing with GNPs during the extrusion process. The milling process was conducted under liquid nitrogen to prevent melting and/or thermal degradation of the polymers. The PE or PP powder was then physically dry-mixed using a high-speed mechanical mixer at 1900 rpm for 5 min. The initial extrusion step involved pre-mixing GNPs with either PP or PE powder at a screw speed of 100 rpm. Subsequently, the pre-dispersed GNPs, referred to as PP+GNP or PE+GNP, were blended with the opposite polymer phase, resulting in the fabrication of (49.37/49.37/1.25% *w*/*w*/*w*. %) PE–PP–GNP composites.

For the blending process, the premixed PE+GNP or PP+GNP composites were manually mixed with the second polymer (PP or PE). These mixtures were then introduced into a Prism ThermoFisher Scientific twin-screw extruder with a diameter of 16 mm and a length-to-diameter (L/D) ratio of 40. Melt compounding was carried out at various screw speeds, specifically 50, 100, and 200 rpm; see Table 2 for the parameters employed during blending.

A temperature profile was applied along the extruder barrel from the feeding zone to the die end and comprised 10 distinct set points of 180 °C, 185 °C, 190 °C, 190 °C, 200 °C, 200 °C, 200 °C, 195 °C, 190 °C, and 180 °C. This controlled temperature regime ensured the optimal melting and mixing of the composite constituents. The resulting molten composite filaments were cooled by passing through a water bath before being pelletised.

Further processing of the compounded pellets involved utilizing a Thermo-Scientific Haake Minijet Pro injection moulding machine. The moulding process was performed using a barrel temperature of 240 °C, a mould temperature of 40 °C, and a pressure of 650 bar with a holding time of 10 s. This procedure yielded disk-shaped samples with a diameter of 25 mm and a thickness of 1.5 mm. These samples were primarily employed for rheological and X-ray diffraction measurements. Moreover, type-V dog bone-shaped specimens (ASTM D638 [31]) were prepared using the same injection moulding machine for subsequent tensile testing.

X-ray diffraction patterns were collected using a PANalytical Empyrean wide-angle X-ray diffraction (WAXD) instrument employing Cu-Kα radiation (45 kV, 40 mA, λ = 1.5419 Å) as the X-ray source. The crystalline structure of the GNPs was studied at 25 °C in the 10–30° 2θ range.

Differential scanning calorimetry (DSC) measurements were carried out using a Mettler Toledo instrument (Columbus, OH, USA) with a carrier gas flow rate of 10 mL/min. Specimens (~7 mg) underwent a controlled heating regime from −50 °C to 200 °C at a heating rate of 5 K/min. Subsequently, the samples were held at 200 °C for 2 min to eliminate thermal history. Following this, the samples were cooled to −50 °C at a rate of 1 K/min. The melt crystallization temperatures (T_c_) were extracted from the cooling curves.

Oscillatory rheology measurements were carried out using an Anton Paar MCR 302 Rheometer (Graz, Austria) equipped with parallel plate geometry set to a gap width of 1 mm. Initially, amplitude sweep tests were executed across a strain range of 0.1–1000% at a temperature of 200 °C and a frequency of 1 rad/s. The objective was to identify the linear viscoelastic range for all materials. Subsequently, the samples underwent a frequency sweep test covering the range 0.1 to 600 rad/s at 200 °C, employing a strain amplitude of 0.1%.

The morphology of selected samples was examined using a Zeiss Sigma field emission scanning electron microscope (Jena, Germany). All samples were cryo-fractured under liquid nitrogen and positioned on a carbon adhesive tape affixed to an aluminium sample holder. Subsequently, all samples were coated with approximately a 10 nm layer of Pd/Pt (Cressington 108 auto, Watford, UK) prior to imaging.

Tensile mechanical testing was performed using an Instron 5800R instrument (Norwood, MA, USA), fitted with a 100 kN load cell. A minimum of five dog bone-shaped specimens (type-V) were prepared and tested for each composite (ASTM D638). The tests were conducted utilizing a video extensometer and maintaining a constant crosshead speed of 10 mm/min.

## 3. Results

### 3.1. Crystallization Behaviour

**Figure 1:**
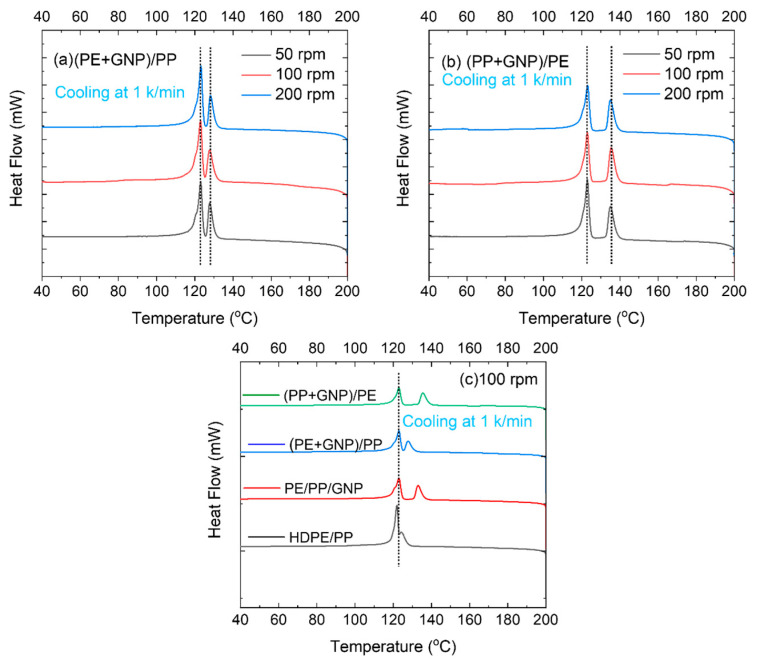
DSC cooling curves (1 K/min.) for composites of (**a**) (PE+GNP)/PP and (**b**) (PP+GNP)/PE, and (**c**) for different blending sequences and a screw speed of 100 rpm at the rate of 1 k/min.

Figure 1 shows the DSC cooling curves for the PE/PP/GNP composites, prepared at the different screw speeds employed. Most notably, the DSC cooling scans show the nucleating effect the GNPs have for both PE and PP and the altering of the crystallization behaviour of both polymers within the composite matrix.

Crystallization peaks were observed for both PE and PP at approximately 122 °C and 124 °C, respectively. The first peak (124 °C) can be ascribed to PP, appearing almost as a shoulder to the sharper, more intense PE peak (122 °C). With the inclusion of 1.25 wt.% GNPs, the crystallization peak (T_c_) of PP shifts to the higher temperature of 133 °C. Interestingly, the crystallization behaviour exhibited distinct variations when GNPs were pre-dispersed in the two different polymer phases before blending. The thermal properties of these composites, obtained from the DSC thermograms, are listed in Table 3. The degree of crystallization was also calculated using Equation (1), as follows:

(1) $$X\%=\frac{{∆}_{Hm}}{{∆}_{Hm}^{100\%}\times \emptyset }$$

where ∅ is the weight fraction of the polymer being investigated, ${∆}_{Hm}$ is the enthalpy of the heat of the fusion of the polymer, and ${∆}_{Hm}^{100\%}$ is the heat of the fusion of a theoretically 100% crystalline polymer taken as 207 and 293 J/g for PP and PE, respectively [32,33,34].

In the case where the GNPs were pre-dispersed within the PE phase before blending with PP, the crystallization peak for PP had a maximum at 127 °C. In contrast, when the GNPs were pre-dispersed within the PP phase, a higher peak at 135 °C was observed. Thermal analysis is useful for assessing particle localization in immiscible polymer blends, as the crystallization peak position and width can reveal the preferential distribution of the GNPs. Previous studies by Cardinaud and McNally on multiwalled carbon nanotubes (MWCNTs) within a polyethylene terephthalate/low-density polyethylene blend (PET/LDPE) and Entezam et al. on nanoclay (Cloisite 30B) in PP/PET blends have demonstrated that selective localization impacts crystallization temperatures, as seen with the increase in T_c_ by 20 °C and 11 °C for PET in the respective studies. This highlights the effect of blending sequence and nucleation dynamics on the crystallization behaviour [35,36].

These studies highlight the role of processing conditions, blending sequence, and nucleation dynamics on the crystallization behaviour. However, these effects are less pronounced in PE, as indicated by its consistent crystallization temperature, due to PE’s rapid crystallization kinetics. It must be noted that the introduction of GNPs induces broader crystallization peaks for PE, accompanied by the evolution of an additional peak at lower temperatures around 120 °C, ultimately contributing to an increased width of the PE crystallization peak, indicating a range of crystallite sizes with variable packing densities [37,38] (Figure 1c).

The minimal impact on PE’s crystallization may be due to limited GNP dispersion and interaction with HDPE chains, reducing GNP participation in the crystallization process. In contrast, GNPs significantly affected the crystalline structure of PP, especially when pre-dispersed within the PP phase.

Varying the screw speed also resulted in some changes in X%, see Table 3. It is clear that when GNPs are premixed with PP, the X% of PP in the final blends increases from approximately 33% at 50 rpm to about 36% at 200 rpm. This observation aligns with the crystallinity obtained from XRD patterns (Table 4), where the overall crystallinity increases from 35% at 50 rpm to approximately 38% at 200 rpm. Conversely, when GNPs are premixed with PE, the X% of PE increases significantly from around 55% at 50 rpm to about 61% at 100 rpm, before dropping again at 200 rpm. This highlights the influence screw speed can have on the degree of crystallinity.

**Figure 2:**
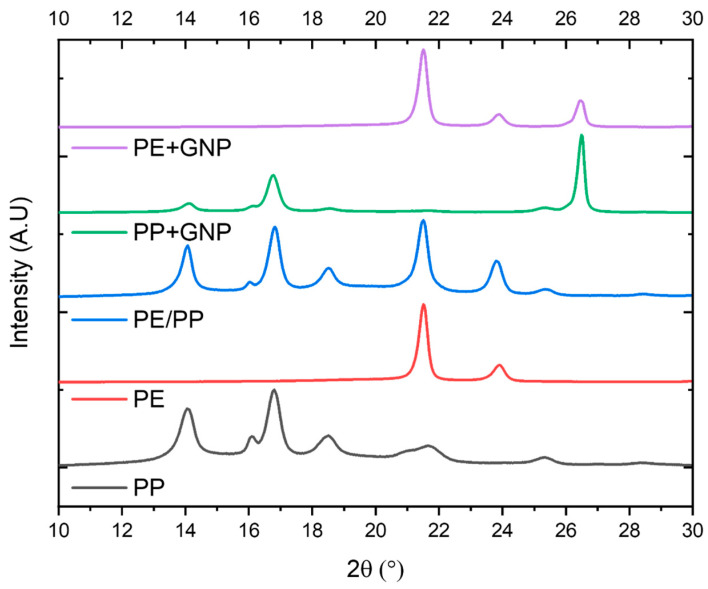
X-ray diffraction patterns of the neat PE, PP, and their composites with 1.25 wt.% GNP loading.

Figure 2 shows the XRD patterns for unfilled PE and PP, and the composites with GNP inclusion into PE and PP.

**Table 4:** XRD parameters for the (PP+GNP)/PE composites extracted from Figure 3.

(PP+GNP)/PE (50 rpm) X% = 35.09%								
2θ	14.00	16.00	16.80	18.53	21.5	23.84	25.36	26.52
Intensity	32,429	11,718	40,971	18,924	90,533	19,182	7136	91,990
FWHM	0.46	1.00	0.49	1.11	0.39	0.56	1.66	0.28
Size (nm)	18.18	8.38	17.12	7.58	21.66	15.15	5.13	30.46
**(PP+GNP-HP)/PE (100 rpm) X% = 38.89%**								
2θ	14.00	16.00	16.75	18.44	21.5	23.75	25.36	26.43
Intensity	33,795	12,443	43,855	20,027	85,992	15,624	7233	74,642
FWHM	0.46	0.97	0.47	0.96	0.38	0.59	1.73	0.27
Size (nm)	18.18	8.64	17.85	8.76	22.23	14.38	4.92	31.58
**(PP+GNP-HP)/PE (200 rpm) X% = 38.80%**								
2θ	14.00	16.00	16.75	18.44	21.5	23.75	25.33	26.43
Intensity	32,640	15,831	38,217	18,470	95,825	15,831	6068	58,840
FWHM	0.42	0.49	0.44	0.96	0.35	0.58	1.45	0.27
Size (nm)	19.91	17.11	19.07	8.76	24.14	14.62	5.87	31.58

**Figure 3:**
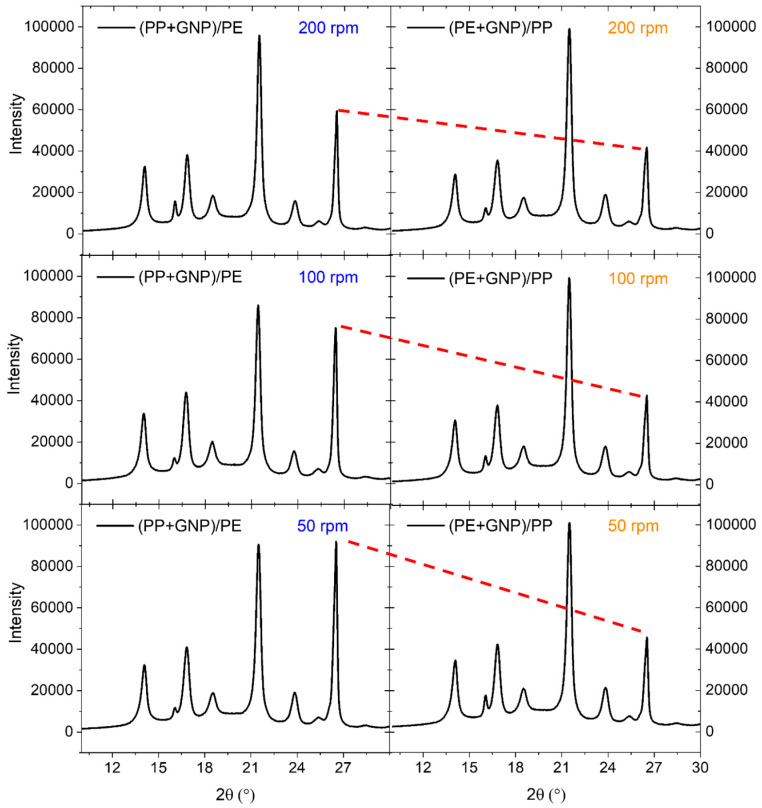
X-ray diffraction patterns of the (PE+GNP)/PP and (PP+GNP)/PE composites prepared at different screw speeds. The red dotted lines are used as guides to show changes in the intensities at 2θ = 26°, associated with the (002) crystalline structure of GNPs, as influenced by blend sequence and screw speed.

Notably, the two prominent reflections at 2θ = 21.5° and 2θ = 23.8°, corresponding to the (110) and (200) crystallographic planes, respectively, are attributed to the crystalline planes of PE [39,40]. Similarly, distinctive peaks were observed at 14.0°, 16.9°, 18.5°, and 25.4° for PP, corresponding to the α-form crystallographic planes (110), (040), (130), and (060), respectively. Moreover, the peak around 21° in the XRD pattern of PP corresponds to the α-form crystallographic plane (131) [41,42]. A very small peak that appeared at 2θ = 16.0° corresponds to the β-form crystallographic plane (300) [41,43]. The diffraction peak around 26.4° is attributed to the (002) plane of graphene within the GNPs [10,38]. It is quite clear that the intensity of this peak at around 26.4° was significantly enhanced upon the inclusion of GNPs in PP. The FWHM of this peak at 2θ = 26.4° was found to be 0.25 in PP+GNP and 0.33 in PE+GNP, indicating a more ordered structure of the GNPs in PP. This observation supports our findings from the DSC data, confirming that the inclusion of GNPs had a minimal effect on the nucleation of PE. This phenomenon may stem from the limited interaction between PE and GNPs, further compounded by a relatively low GNP concentration and a very low effective surface area to induce crystallization. This effect aligns with previous research, where similar outcomes were noted, and such behaviour is attributed to the formation of GNP aggregates that function as physical impediments retarding nucleation [44,45].

The crystalline structures of the (PE+GNP)/PP and (PP+GNP)/PE composites were studied further using X-ray diffraction (XRD) (Figure 3). The XRD patterns show differences in crystallization behaviour arising from the two distinct blending sequences and the influence the blending strategy had on the crystallinity of the final composites. Distinct peaks at 2θ values of 14°, 16.85°, 18.51°, 21.5°, 23.85°, and 26.4° can be observed.

Considering that the majority of GNP layers are confined within the respective PE and PP phases in the premixed (PE+GNP)/PP and (PP+GNP)/PE composites, the interaction and distribution of GNPs within these phases substantially influence the intensity of the (002) crystalline structure of GNP at 2θ = 26°. Consequently, the XRD patterns reveal a sharper peak at 2θ≈26° in (PP+GNP)/PE composites, where GNPs and PP are pre-dispersed prior to blending, in contrast to the (PE+GNP)/PP composites (see dotted line in Figure 3). This more intense peak, having a smaller full width at half maximum (FWHM) values (see Table 4 and Table 5), suggests a more ordered arrangement of graphene layers with diminished disorder or defects [46]. The values of the intensities of the peaks, FWHM, and the 2θ positions of the (PP+GNP)/PE and (PE+GNP)/PP composites at different screw speeds are shown in Table 4 and Table 5, respectively.

The (002) peak at 2θ ≈ 26° also holds a direct correlation with the interlayer spacing between the graphene layers within the graphite crystal. Notably, the interlayer spacing in this context is relatively expansive compared to densely stacked graphite, resulting in a sharper and narrower (002) peak in the XRD pattern. This manifestation of a smaller FWHM denotes enhanced structural alignment. Conversely, a broader (002) peak, characterized by a larger FWHM, signifies greater disorder or imperfections in the graphene layers, corroborated by the DSC results [47,48]. The narrowing of the peak arises from the reduced interlayer spacing owing to stronger van der Waals interactions between the graphene layers, culminating in a broader and less sharp (002) peak in the XRD pattern, leading to a larger FWHM. 

Furthermore, smaller FWHM values lead to larger crystal sizes according to the following Scherrer equation as shown in [49]:

(2) $$L=\frac{0.9\lambda }{\beta \;\cos\theta }$$

where λ is the wavelength (1.5419 Å), β is the FWHM of the peak, and θ is the peak intensity. It is interesting to note that the intensity of the peak associated with the β-form crystallographic plane (300) at 2θ = 16° in (PP+GNP)/PE blends gradually increases (Figure 3 and Table 4) as screw speed increases indicating that, as screw speed increases, the β-crystals are developing. A similar trend for the α-crystals can be observed in the (PP+GNP)/PE composite. Again, this result is in agreement with the DSC results where PP displayed accelerated crystallization and an enhanced degree of crystallinity with increasing screw speed. Further, using Gaussian fitting, the degree of crystallinity from the XRD curves can be also calculated as follows [50]:

(3) $$X=\frac{A_{c}}{A_{c}+A_{a}}\times 100\%$$

where $A_{c}$ is the area under the crystalline peaks and $A_{a}$ is the area under the amorphous curve. The values listed in Table 4 show that the degree of crystallinity in (PP+GNP)/PE blends increases with screw speed, mirroring the trends seen for PP X% from DSC measurements. This indicates that the X% is primarily governed by the change in the crystallinity of PP in these blends. On the other hand, the degree of crystallinity in (PE+GNP)/PP blends remained somewhat the same for all screw speeds, but with similar values to the crystallinity of (PP+GNP)/PE blends at higher screw speeds (100 and 200 rpm).

### 3.2. Tensile Properties

**Figure 4:**
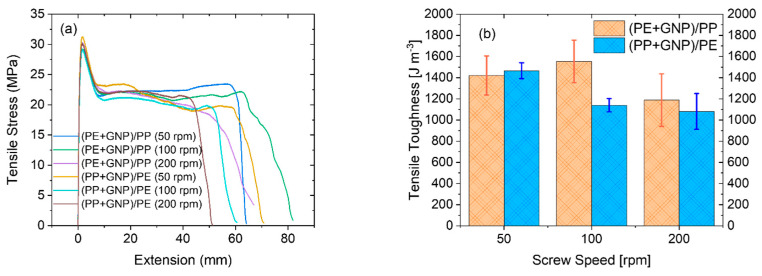

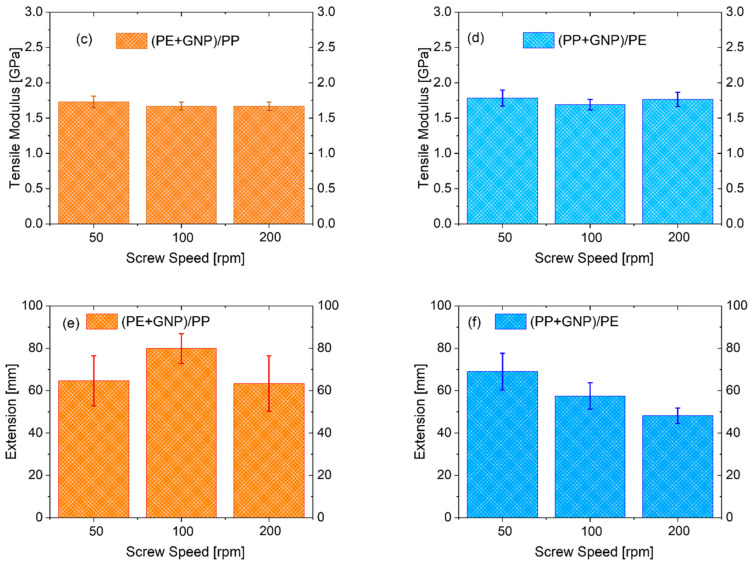
(**a**) Representative tensile stress–strain curves of the composites and variation in (**b**) tensile toughness, (**c**,**d**) tensile modulus of the (**c**) (PE+GNP)/PP and (**d**) (PP+GNP)/PE blends, and (**e**,**f**) tensile extension of the (**e**) (PE+GNP)/PP and (**f**) (PP+GNP)/PE composites measured for the different screw speeds.

Given the effect the inclusion of GNP has on the crystallization behaviour of the PE/PP blend systems, the tensile mechanical properties of the two composite systems, (PE+GNP)/PP and (PP+GNP)/PE, were measured and plotted as a function of screw speed, see Figure 4. The results indicate that the different blending sequences employed have a marginal effect on the tensile moduli of the composites. However, there is some variation in the elongation at break and tensile toughness, where the (PP+GNP)/PE composite exhibits a reduction in toughness (Figure 4b) and elongation at break (Figure 4f) by ~25% and ~28%, respectively, relative to the (PE+GNP)/PP counterpart (Figure 4e) at 100 rpm. Tensile toughness is commonly described as the energy a material can absorb before breaking. In other words, it is the area under the stress–strain curve obtained from a tensile test. Thus, tensile toughness is determined by calculating the integral of the area under the stress–strain curves [51].

The elongation at break of (PP+GNP)/PE decreased by 28% compared to (PE+GNP)/PP mixed at 100 rpm. Further insights from the ANOVA indicated that the results for (PP+GNP)/PE at different screw speeds (Figure 4f) are statistically significant; in contrast, the results for (PE+GNP)/PP at different screw speeds (Figure 4e) are not. This indicates that changing screw speed may not be a determining factor when GNP is pre-dispersed in the PE polymer. This trend aligns well with the conclusions made from the measurement of crystallinity from both DSC and XRD. Changes in the degree of crystallinity from XRD measurements support these findings where the overall crystallinity increases in (PP+GNP)/PE blends when screw speed increases but the crystallinity of (PE+GNP)/PP remains the same at ~38%. Specifically, composites characterized by more intense crystalline structures, as exemplified by (PP+GNP)/PE blends, are anticipated to be less ductile, as blends having greater crystalline regions inherently tend to be more brittle. It can also be seen that the enhanced crystallinity for PP in (PP+GNP)/PE resulted in a reduction in elongation at break (Figure 4f) and tensile toughness (Figure 4b). In contrast, the lowest crystallinity of PP (X% = 27% Table 3) was recorded for the (PE+GNP)/PP blend at a screw speed of 100 rpm, where, surprisingly, the highest elongation at break (80 ± 7.00 mm) (Figure 4e) and tensile toughness (1553.8 ± 201.47 J m^−3^) (Figure 4b) were obtained, with values very close to the tensile toughness of the neat blend (1577 ± 283 J m^−3^). This indicates that the ductility and toughness of the blends are governed by the crystallinity of PP due to GNP localization in the PP phase and screw speed. However, not all changes in crystallinity significantly altered the overall stiffness of the final composites. This could be due to the overall degree of crystallinity, which remained somewhat constant, indicating that the stiffness of PE dominates the overall modulus of the blends. Table 6 summarizes the tensile properties of composites of polyolefins with GNPs or their derivatives, such as graphene oxide (GO).

From Table 6, it can be inferred that the mechanical properties are also affected by the characteristics of GNPs, including their lateral size and number of layers. Furthermore, by employing blending sequence strategies, it is possible to maintain tensile modulus and strength superior to those of single polymer composite systems, such as PP/GNP or PE/GNP at low loadings, while also improving tensile elongation.

### 3.3. Morphological Evolution

**Figure 5:**
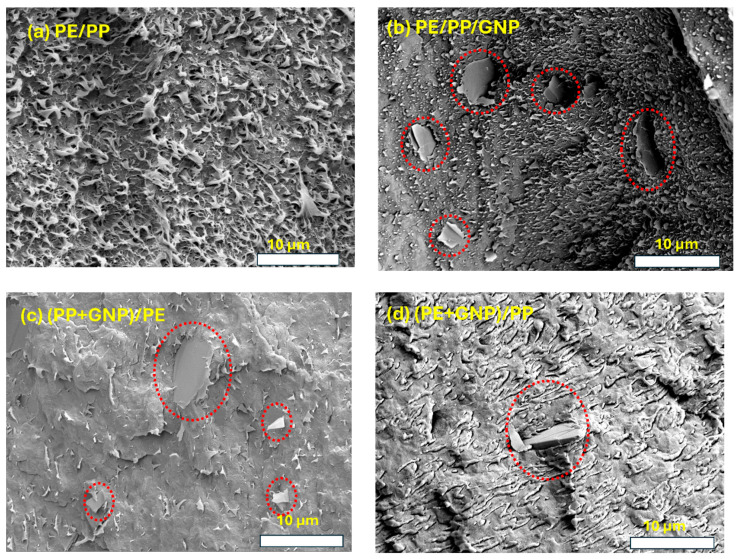
SEM images of the (**a**) neat (PE/PP), (**b**) PE/PP/GNP, (**c**) (PP+GNP)/PE, and (**d**) (PE+GNP)/PP composites extruded at the speed of 100 rpm. Scale bars are 10 µm in all images. Red circles show the GNPs dispersed within the blends.

The extent of GNP dispersion and distribution in each polymer and blend was examined using scanning electron microscopy (SEM). Images of each blend prepared using the different blending sequences at 100 rpm were recorded and compared to the neat blend (Figure 5). The neat blend showed a pseudo co-continuous structure (Figure 5a), while the composites exhibited different morphologies. GNPs are shown within the red circles in the images. Simultaneously blended composites (Figure 5b) showed a similar morphology to that of the neat blend with randomly distributed GNPs within the blend. The (PP+GNP)/PE composite has a smooth surface with differently sized GNP platelets randomly dispersed in the composite matrix, indicating some degree of GNP breakage during blending. The smooth surface is also an indication of a more brittle blend, in agreement with the mechanical properties reported for this blend (see Figure 4). However, the (PE+GNP)/PP has a more ductile surface with evidence for the formation of an elongated co-continuous phase, again in agreement with the highest elongation and tensile toughness values reported in Figure 4. In such a scenario, when force is applied to this type of morphology, the PP phase can stretch without causing slippage in the interphase regions. Therefore, this type of morphology exhibits a failure mechanism like crazing in rubber-filled blends. This morphology facilitates the occurrence of crazing in the PP phase, where the formation of fibrils and elongated structures enhances blend elasticity. The fibril structures resulting from the crazing phenomenon, visible in SEM images (Figure 5d), act as reinforcing elements that absorb and dissipate energy, distribute stress more evenly, collectively contributing to the improved tensile toughness (Figure 4b) of this blend.

It is certain that the mixing sequence has created different morphologies. This highlights the significant role of the blending strategy, as it results in completely different outcomes despite having the same composition and filler loading. Consequently, this difference in morphology and crystallization behaviour results in varying mechanical properties, as discussed earlier.

### 3.4. Rheological Properties

**Figure 6:**
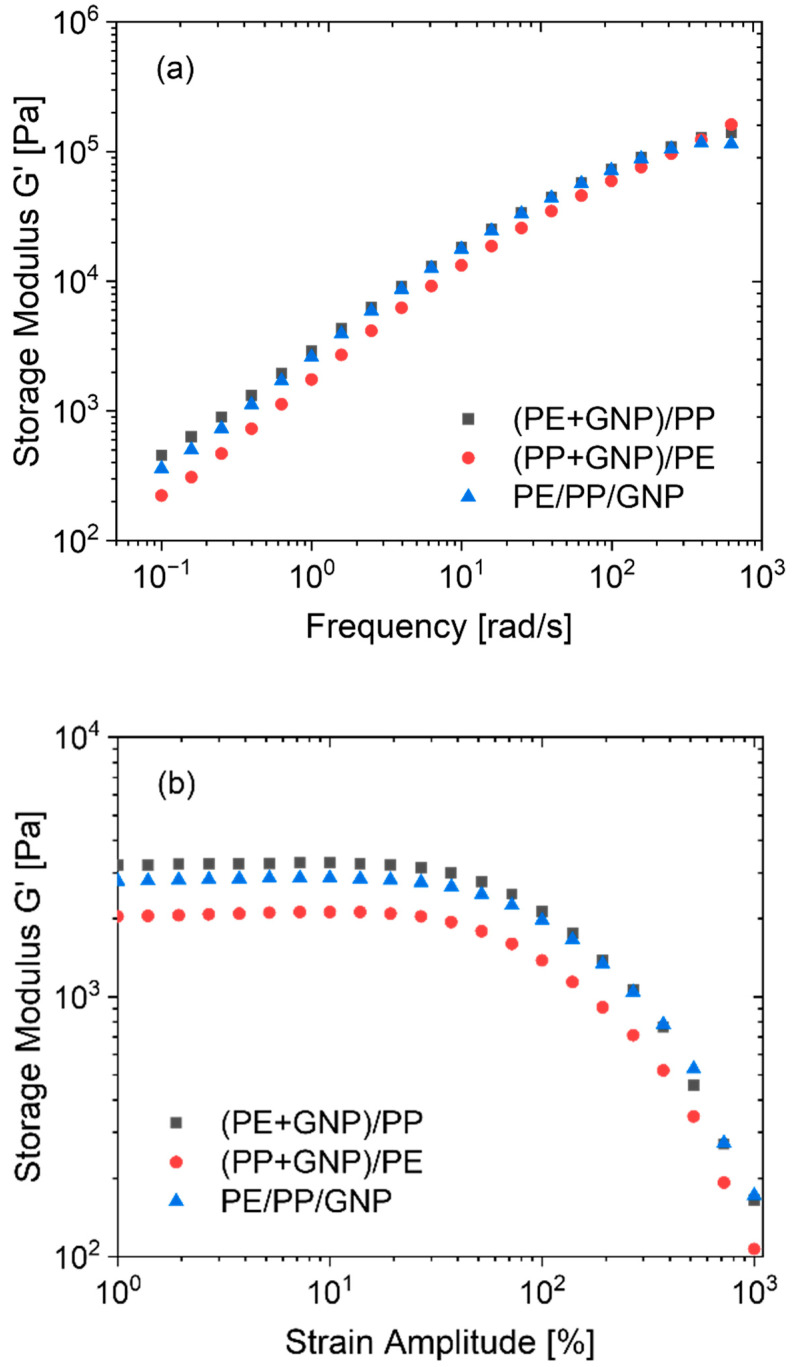
(**a**) Small and (**b**) large amplitude oscillatory shear test responses of the three different composites prepared by melt mixing at 100 rpm. The tests were carried out under nitrogen and at 200 °C.

Now that the changes in morphology influenced by the blend sequence have been confirmed, the effects of blend sequence on the rheological properties in the molten state were investigated. Figure 6 shows the variation in the storage moduli of the blends prepared via the different sequences at a screw speed of 100 rpm as a function of angular frequency (Figure 6a) and strain amplitude (Figure 6b). The above results demonstrate how the GNP is more compatible with PP, accelerating the crystallization rate of PP. Additionally, it was shown how the selective localization of GNP in PP can produce composites that are significantly less tough (Figure 4b). Furthermore, differences in rheological properties also become apparent. It is evident that blends with a co-continuous morphology, i.e., (PE+GNP)/PP and PE/PP/GNP, which are also tougher than (PP+GNP)/PE, exhibit higher moduli compared to the blend where GNP was initially mixed in the PP phase. Nonetheless, it is pertinent to note that melt properties are influenced by overall morphologies, rather than localized crystallinities, particularly considering that operating temperatures well exceed the crystalline melting temperatures of both PP and PE (200 °C).

Conversely, for the blend where GNPs and PP were pre-mixed, (PP+GNP)/PE, relatively lower modulus values were recorded. This is attributed to the phase-separated morphology where no co-continuity is observed. While GNPs may display enhanced interaction with PP, they concurrently elevate the viscosity of the PP host phase. This increased viscosity alters the viscosity ratios of the blends. In contrast, the higher viscosity of the PE+GNP phase effectively immobilizes the neat PP phase, allowing the PP phase to distribute more evenly within a co-continuous structure. It is well-established that blends with co-continuous morphologies exhibit elevated melt elasticities compared to their sea-island morphology counterparts [35,58,59]. Consequently, these rheological variations provide an additional lens through which the divergent composite characteristics can be discerned.

Increasing the viscosity of the PP phase disrupts the viscosity balance of the blend. On one hand, increasing GNP in the PP phase makes the PP phase brittle and reduces the adhesion between the two phases. On the other hand, when the PE phase is filled with GNP, it significantly increases the viscosity of PE. This results in a greater viscosity difference between the two phases, where the PP phase is surrounded by the stiffer and more viscous PE phase.

## 4. Conclusions

The study highlights the significance of processing conditions in tailoring the properties of composites of PE and PP reinforced with GNPs. The sequence of blending, as well as the interaction between GNPs and the polymer phases, influenced crystalline structure, mechanical behaviour, and blend morphology. The rate of crystallinity, determined from DSC measurements, and the crystalline structures from XRD proved to be the most responsive parameters to processing variations, whereas tensile modulus remained unchanged. However, elongation at break and tensile toughness were more responsive to the changes. The elongation at break of the (PP+GNP)/PE blend decreased by 30% on increasing the screw speed from 50 rpm to 200 rpm. In addition, a 28% reduction in elongation at break was also obtained for the (PP+GNP)/PE blend prepared at 100 rpm compared to the (PE+GNP)/PP blend processed at the same screw speed. The blend with GNPs premixed within the PE phase had higher elongation at break values and was tougher due to their unique morphology. Blends with ductile morphologies, i.e., (PE+GNP)/PP and PE/PP/GNP exhibited higher elastic moduli in the melt state at 200 °C.

This comprehensive study highlights the dynamic relationship between processing, blend morphology, and performance in composites of polymers and GNPs, providing valuable insights and a route to optimizing blend properties. Here, the selection of blend ratio and GNP concentration was a deliberate choice for establishing a model blend for fundamental studies. Further research necessitates investigating supplementary processing parameters, optimizing GNP concentration, and exploring blend composition to unlock the full potential of these advanced materials.

## Figures and Tables

**Table 1:** Specifications of the GNPs used in this study [30].

Nanene-002 Technical Specifications				
Property	Measurement			Method
Layers ≤ 5	18%			Raman
Layers ≤ 10	73%			Raman
Layers 10–100	27%			Raman
Defect Ratio	0.3AV. I_D_/I_G_			Raman
Lateral Dim.	<26.5 μm			SEM
**Concentration (At. %)**				
Carbon	Oxygen	Fluorine	Sulphur	Nitrogen
96 ± 3.0	3.0 ± 2.0	1.0 ± 1.0	1.0 ± 1.0	0.3 ± 0.3

**Table 2:** Extrusion parameters used for preparing the PE- or PP+GNP composites.

Premixed	Second Polymer	Feeding Rate (g/min)	Screw Speed (rpm)	Residence Time (s)
PE+GNP	PP	12.65	50	148
PE+GNP	PP	12.65	100	89
PE+GNP	PP	12.65	200	57
PP+GNP	PE	14.07	50	144
PP+GNP	PE	14.07	100	88
PP+GNP	PE	14.07	200	50

**Table 3:** Thermal properties acquired from DSC scans. Heating at 5 and cooling at 1 k/min.

	T_m (PE)_ °C	T_m (PP)_ °C	${∆H}_{m}$ _(PE)_ J/g	${∆H}_{m}$ _(PP)_ J/g	T_c (PE)_ °C	T_c (PP)_ °C	X% _(PE)_	X% _(PP)_
(PP+GNP)/PE (50 rpm)	133	165	79	34	123	135	55	33
(PP+GNP)/PE (100 rpm)	131	166	70	37	123	136	49	36
(PP+GNP)/PE (200 rpm)	132	165	71	37	123	135	49	36
(PE+GNP)/PP (50 rpm)	131	164	80	33	123	128	55	32
(PE+GNP)/PP (100 rpm)	133	165	89	28	123	128	61	27
(PE+GNP)/PP (200 rpm)	133	167	79	32	123	128	55	32

**Table 5:** XRD parameters for the (PE+GNP)/PP composites extracted from Figure 3.

(PE+GNP)/PP (50 rpm) X% = 38.12%								
2θ	14.00	16.00	16.83	18.51	21.5	23.84	25.39	26.51
Intensity	30,800	15,345	37,971	18,321	92,208	18,691	5996	40,443
FWHM	0.45	0.53	0.49	1.16	0.38	0.56	1.65	0.30
Size (nm)	18.58	15.82	17.12	7.25	22.23	15.15	5.16	28.43
**(PE+GNP)/PP (100 rpm) X% = 38.26%**								
2θ	14.00	16.00	16.81	18.51	21.5	23.84	25.39	26.51
Intensity	31,038	13,857	38,330	18,475	99,694	18,404	6112	42,675
FWHM	0.46	0.53	0.49	1.34	0.38	0.58	1.60	0.30
Size (nm)	18.18	15.82	17.12	6.28	22.23	14.63	5.32	28.43
**(PE+GNP)/PP (200 rpm) X% = 38.46%**								
2θ	14.00	16.00	16.81	18.51	21.5	23.84	25.37	26.50
Intensity	28,721	12,619	35,406	17,443	99,480	18,904	5894	41,828
FWHM	0.47	0.67	0.51	1.33	0.38	0.57	2.47	0.33
Size (nm)	17.79	12.51	16.45	6.32	22.23	14.88	3.44	25.84

**Table 6:** Mechanical properties of representative polyolefin composites with GNPs obtained from tensile tests, comparing results from recent literature to those from the current study.

Composite	Filler Content (wt.%)	Tensile Modulus (GPa)	Tensile Strength (MPa)	Elongation at Break (% Change) ^a^	Reference
PP/GH GNP	1	1.54 ± 0.04	23.3 ± 0.3	−88.33	[52] ^b^
PP/GM GNP	1	1.65 ± 0.04	27.7 ± 0.9	−86.91	[52]
PP/GNP	1	1.70 ± 0.10	32.1 ± 0.6	−95.01	[53]
PE/GNP	10	0.933	23	N/A	[54]
PE/M5 GNP	1	0.45 ± 0.01	19.13 ± 0.3	−36.44	[55] ^c^
PE/M15 GNP	1	0.53 ± 0.01	21.23 ± 0.2	−27.76	[55] ^c^
PE/M25 GNP	1	0.54 ± 0.01	21.55 ± 0.2	−90.14	[55] ^c^
PE/PP/GO	2	1.18 ± 0.1	25.1 ± 1.4	−12.5	[56]
(Graphene + PP)/PE	7	1.29 ± 0.14	19.1 ± 2.4	−14.28	[28]
(Graphene + PE)/PP	7	0.69 ± 0.13	24.6 ± 2.2	+132.14	[28]
Graphene/PE/PP	7	0.97 ± 0.36	17.2 ± 3.7	+42.85	[28]
R-(PE/PP)/FLG	1	1.46 ± 0.04	23.39 ± 0.2	−64.58	[57] ^d^
(PE+GNP)/PP	1.25	1.67 ± 0.05	29.17 ± 0.4	+16.26	Current Work ^e^
(PP+GNP)/PE	1.25	1.69 ± 0.07	29.97 ± 1.2	−16.38	Current Work

^a^ Change is relative to the unfilled polymer here. + and − signs indicate an increase and decrease, respectively. ^b^ GH GNPs have a thickness of 15 nm, while GM GNPs have a thickness of 6–8 nm. ^c^ GNPs M5, M15, and M25 represent average platelet diameters of 5 μm, 15 μm, and 25 μm, respectively. ^d^ In this study, a mixed sourced recycled R-(PE/PP) was used with few-layer (6–10) graphene FLG powder. ^e^ We considered the results of 100 rpm in this table.

## Data Availability

The original data presented in the study are included in the article, further inquiries can be directed to the corresponding author.
